# Impact of Conjunctivochalasis on Visual Quality of Life: A Community Population Survey

**DOI:** 10.1371/journal.pone.0110821

**Published:** 2014-10-20

**Authors:** Qihua Le, Xinhan Cui, Jun Xiang, Ling Ge, Lan Gong, Jianjiang Xu

**Affiliations:** 1 Department of Ophthalmology, Eye & ENT Hospital of Fudan University, Shanghai, China; 2 Research Center, Eye & ENT Hospital of Fudan University, Shanghai, China; 3 Myopia key laboratory of ministry of health, Eye & ENT Hospital of Fudan University, Shanghai, China; 4 Department of Ocular Surface Disease, Shanghai Eye Disease Prevention & Treatment Center, Jing’an District, Shanghai, China; Boston University School of Medicine, United States of America

## Abstract

Conjunctivochalasis (Cch) is a very common ocular disorder, which can cause an unstable tear film and ocular discomfort. The study of vision-related quality of life (VR-QoL) in a community population with Cch can provide a better understanding of the impact of Cch on common people than objective clinical examinations alone. This cross-sectional comparative study enrolled 360 participants ≥40 years old living in Sanle Community, Shanghai. In the study, 198 subjects were diagnosed with Cch and 86 with dry eye syndrome (DES) without Cch. The remaining 76 subjects were normal controls. Socio-demographical data were collected, and Cch and related ocular symptoms and signs were evaluated. In addition, all participants were required to complete the Chinese version of the 25-item National Eye Institute Visual Functioning Questionnaire (NEI VFQ-25) and Ocular Surface Disease Index Questionnaire (OSDI). Main outcome measures include the comparison on the OSDI score and VFQ-25 score among the subgroups, and the correlation of these scores with the socio-demographical and clinical data. The results revealed that subjects with Cch had significantly decreased tear film stability even compared with those with DES (P = 0.001). The participants with either Cch or DES reported significantly higher OSDI scores and lower VFQ-25 composite scores than the normal controls (P<0.001 and 0.007 respectively). Further comparisons among the subgroups of Cch revealed that the following factors were associated with higher OSDI scores and lower VFQ-25 composite scores: nasal-side Cch, chalasis folds higher than tear meniscus height, punctal occlusion, or increased extent of chalasis on digital pressure. In conclusion, Cch was associated with an adverse impact on VR-QoL in a community population, and the impairment in VR-QoL had a significant correlation with disease severity and tear film abnormalities.

## Introduction

Conjunctivochalasis (Cch), originally described by Hughes [1], is characterized by a loose, redundant, nonedematous inferior bulbar conjunctiva, which is typically interposed between the globe and the lower eyelid. Its pathogenesis is thought to be associated with loss of conjunctival epithelial cohesiveness and increased collagenolytic activity [2], [3]. The condition can be localized in the nasal, central, or temporal part of the lower eyelid and usually occurs bilaterally [1], [4]. Although Cch is a very common ocular disorder, with a prevalence of 44.1% in the Chinese population [5], it is often overlooked or may be considered as a normal senile change. However, Cch has been considered as the the precursor to pterygium [6], and has a close relationship with contact lens wear [7] and refractive error, especially hyperopia [8]. Moreover, findings on dry eye syndrome (DES) and its pathogenesis in recent years have focused attention on the relationship between Cch and DES [9]. Cch has been reported to be an important factor in disturbing tear outflow, making the tear film unstable and increasing the tear osmolarity, consequently causing ocular discomfort such as irritation, tearing and blurred vision [2], [10]. Moreover, surgical treatment for Cch can significantly improve tear stability and clinical dry eye symptoms [11].

In recent years, the importance of ocular disorder-associated impairments in quality of life has been increasingly recognized. Quality of life measures refer to additional components of a disease such as patient-reported physical symptoms and social/role, psychological/emotional, and cognitive functioning data, which objective clinical examinations cannot provide. Many studies have demonstrated that DES frequently causes fluctuations or reductions in the quality of vision [12], further leading to an adverse impact on vision-related quality of life (VR-QoL) [13]–[14]. Nevertheless, the impact of Cch on VR-QoL has been somewhat neglected because it is usually less symptomatic than DES and tends not to be a common cause of permanent visual morbidity compared with other ocular diseases, such as cataract, glaucoma and age-related macular degeneration.

Although Cch rarely causes severe vision-threatening problems, its prevalence increases dramatically with age [5], [15]. Given projections of longer life expectancy in developed areas of China, such as Shanghai, the importance of VR-QoL in subjects with Cch can be expected to increase. Thus far, no studies have been performed previously to address this issue. Therefore we conducted a cross-sectional study in a community population to investigate the impact of Cch on VR-QoL and explore its associated factors.

## Subjects and Methods

### Study population and socio-demographic data collection

The study was conducted between March 15, 2010, and June 30, 2010. It was approved by the ethics committee of Eye & ENT Hospital of Fudan University and executed according to the tenets of the Declaration of Helsinki. A total of 360 residents living in Sanle Community were randomly selected and enrolled in this study, all of whom were ≥40 years old. Sanle community is located in Jing’an district, the center region of Shanghai, China. This community was selected due to its metropolitan location, population stability and the support by local government and medical institution.

**Figure 1:**
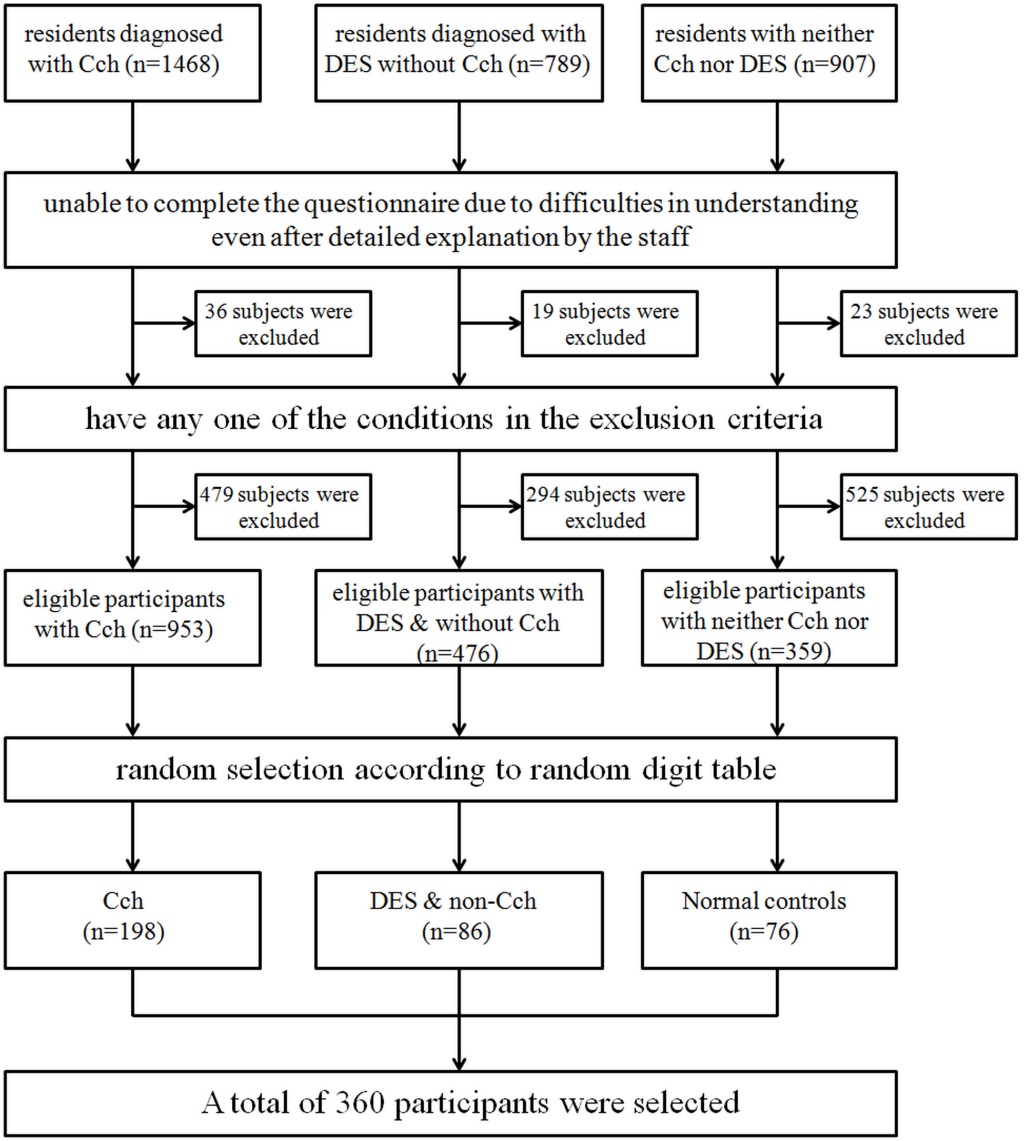
The protocol for the study population selection is shown here.

Subjects with the following abnormalities were excluded: blood pressure higher than 160/95 mmHg, diabetes mellitus, autoimmune or other systemic disorders, corneal disorders which would probably affect visual acuity such as corneal leukoma, pterygium and corneal ulcer, the intraocular pressure(IOP) higher than 21 mmHg or lower than 9 mmHg, lens opacity greater than grade II according to Lens Opacity Classification System III (LOCSIII), any abnormalities in the fundus photograph, refractive error more than +/−6D, and the history of contact lens wear, allergic conjunctivitis or ocular surgery. Figure 1 presented the selection for the study population. Written informed consent was obtained from all the enrolled participants.

Before the questionnaire surveys and the ocular examination, the socio-demographic data were collected from all the subjects, including age, sex, occupation, educational level, marital status, history of systemic disorders, especially autoimmune disorders, smoking habits, alcohol intake, daily time spent using a computer and watching television, and daily exposure to air conditioning. The subjects were also questioned about common symptoms that occur in DES, including a burning sensation, itching, foreign body sensation, sensation of stabbing pain, dryness, photophobia, or asthenopia.

### Questionnaire Survey

The Ocular Surface Disease Index (OSDI), developed by Allergan, Inc., is a 12-item patient-reported outcome questionnaire designed to quantify ocular disability due to DES. The Chinese version of OSDI, which has been validated [16] and accepted by many Chinese ophthalmologists [16]–[19], was used in this study. In brief, subjects were questioned with three different subscales: ocular symptoms (e.g., “Eyes that feel gritty?”), vision-related functions and limitations (e.g., “Have problems with your eyes limited you in reading?”), and environmental triggers (e.g., “Have your eyes felt uncomfortable in windy conditions?”) during a 1-week recall period. Each answer was scored based on the frequency of symptoms using a 5-point scale from 0 (indicating no problem) to 5 (indicating a significant problem). The responses to all questions were combined to give a composite OSDI score ranging from 0 to 100, with higher scores indicating more severe symptoms.

A Chinese version of the 25-item National Eye Institute Visual Function Questionnaire (NEI VFQ-25), which was used in previous reports [20]–[22], was administered to all the enrolled subjects. Since the response rate to No.14 item was rather low in the Chinese population [20], we selected item A8 from the NEI-VFQ39 to serve as the appendix of No.14 item according to the instructions of the NEI VFQ-25 manual. If the statistical analysis showed a low response rate to No. 14 item, the result of its appendix would be used so as to alleviate the impact of a high miss rate of No.14 item on the validity and reliability of whole questionnaire. Each item in the questionnaire was assigned to one of the 12 subscales: general health, general vision, ocular pain, near activities, distance activities, social functioning, mental health, role difficulties, dependency, driving, color vision, and peripheral vision. Answers to each question on the VFQ-25 were converted to a 100-point scale, in which 100 represents the best possible score or the minimal subjective impairment, and 0 represents the worst or the maximal. Guidelines published by the NEI were adhered to when calculating the above scale conversions and subscale scores.

### Ophthalmic examinations

After demographic data collection and accomplishment of the questionnaires, all the participants underwent a thorough ophthalmic examination to exclude any ocular abnormalities other than Cch or DES that could potentially affect VR-QoL. The examination included best-corrected visual acuity (BCVA), slitlamp biomicroscopy, direct ophthalmoscopy, fundus photography, tonometry, an optometric examination, and Fourier-domain optical coherence tomography (FD-OCT). All the equipments were transported to a local medical unit for the convenience of the participants.

One experienced corneal specialist, who was masked to dry eye information in the questionnaire, performed slitlamp biomicroscopy examination to assess the grade of Cch. FD-OCT (RTVue-100, Optovue Inc, CA) equipped with a CAM-long (CAM-L) lens was used in the study to assist the evaluation of the severity of Cch [23]. A scan angle of 90 degrees and a vertical beam length of 6 mm were used and the examination was performed at temporal, central, and nasal lower eyelid.

The BCVA was evaluated with a LogMAR visual acuity chart. Tear break-up time (TBUT) and corneal fluorescein staining (CFS) were assessed during slitlamp biomicroscopy examination, as previously described [17], [24]. In brief, a sterile fluorescein strip (Tianjin Jingming New Technological Development Co., Ltd., Tianjin, China), which was slighted moistened with sterile saline, was applied to the inferior fornix and the participant was asked to blink several times. The tear film was then examined with the aid of the cobalt blue filter on the slit lamp while the participant hold the eyes open, refraining from blinking. TBUT refers to the time that elapses before the first dry spot or line appears in the corneal fluorescein layer. TBUT was repeated three times for each eye, and the average time was recorded. To evaluate the severity of CFS, the cornea was divided into five parts: central, nasal, temporal, superior and inferior. The fluorescence staining dots in each part were scored according to Oxford scale [25], and total CFS score was calculated. A noncontact tonometer rather than a Goldmann applanation tonometer was used to measure the intraocular pressure (IOP) so as to avoid any impact on the corneal epithelium or on the fluorescence staining. For the same purpose, Schirmer I test (ST) was finally performed when the rest examinations were all finished.

### Grading system of Cch and diagnostic criteria of DES

Cch was diagnosed and graded according to the following classification proposed by Meller and Tseng [4] as follows: grade 0 (G0, no persistent fold), grade 1 (G1, a single, small fold), grade 2 (G2, two or more folds, but not higher than the tear meniscus), and grade 3 (G3, multiple folds and higher than the tear meniscus). For those diagnosed as G1 or G2, the location of Cch was further specified as T, M, or N, denoting the temporal, middle (or inferior to the limbus), or nasal part of the lower lid, respectively. In addition, the extent of Cch, gaze-dependent changes, and digital pressure–dependent changes were classified, as presented in Table 1.

**Table 1:** Additional grading criteria of Cch.

	1	2	3
Folds versus Tear Meniscus Height (H)	〈tear meniscus	= tear meniscus	>tear meniscus
Punctal Occlusion (O)	without punctal occlusion	with punctal occlusion	
Changes in Down Gaze (DG)	no difference	height/extent of chalasis increases in downgaze	height/extent of chalasis decreases in downgaze
Changes by Digital Pressure (P)	no difference	height/extent of chalasis increases on digital pressure	height/extent of chalasis decreases on digital pressure

The subjects were diagnosed as DES if they had frequent or sustained occurrences of any one of the symptoms mentioned above for at least 3 months, and simultaneously had any two of the following three conditions: (1) ST value of less than 10 mm/5 min; (2) TBUT of less than 10 sec; (3) CFS score≥1.

### Statistical Analysis

Statistical analysis was performed using SPSS 15.0 for Windows (SPSS, Inc. Chicago, IL). Either Mann-Whitney U test or a nonparametric analysis of variance (ANOVA) was applied to compare the age, BCVA, IOP, OSDI scores and NEI VFQ-25 scores among the subgroups. The gender distribution, marital status, occupation, ST and TBUT were analyzed with a chi square test. Multiple regression analysis was performed to evaluate the relationship between clinical variables and the OSDI score. The same analysis was also performed to determine the related factors of the VFQ-25 composite score and subscale scores. A P value less than 0.05 was considered statistically significant.

## Results

A total of 144 men and 216 women were enrolled, with an average age of 63.6±11.9 (range: 41–92). Among them, 198 subjects were diagnosed with Cch(G1,G2, and G3) and 86 with simple DES without Cch(G0). The remaining 76 participants were normal controls who had neither Cch nor DES. There were no significant differences in the gender distribution and in the average age of the three groups. The majority participants were retired and had only completed compulsory education. Other socio-demographic data of the three groups were shown in Table 2.

**Table 2:** Socio-demographic data of enrolled subjects.

	Cch (n = 198)	DES & non-Cch (n = 86)	normal controls (n = 76)	P value
**gender**				0.381
male	75 (37.9%)	32 (37.2%)	37 (48.7%)	
female	123 (62.1%)	54 (62.8%)	39 (51.3%)	
**age**				0.549
average	64.6±11.6	62.8±11.4	61.5±9.6	
range	41–92	41–88	41–85	
**marital status**				0.376
married	163 (82.3%)	64 (74.4%)	56 (73.7%)	
single	32 (17.7%)	22 (25.6%)	20 (26.3%)	
**occupation**				0.013 *
employed	38 (19.2%)	29 (33.7%) ^a^	19 (25.0%)	
unemployed	3 (1.5%)	3 (3.5%)	2 (2.6%)	
retired	157 (79.3%)	54 (62.8%)	55 (72.4%)	
**education**				0.003 †
illiterate	8 (4.0%)	2 (2.3%)	4 (5.3%)	
primary school	28 (14.1%)	10 (11.7%)	8 (10.5%)	
middle school	130 (65.7%)	66 (76.7%)	51 (67.1%)	
college or above	32 (16.2%)	8 (9.3%)^ b^	13 (17.1%)	
**smoking habits**				0.486
non-smokers	169 (85.4%)	74 (86.0%)	69 (90.8%)	
smokers	29 (14.6%)	12 (14.0%)	7 (9.2%)	
**alcohol intake**				0.533
non-drinkers or abstainers	168 (84.8%)	72 (83.7%)	68 (89.5%)	
drinkers	30 (15.2%)	14 (16.3%)	8 (10.5%)	
**use of computer**				0.024 *
>3 h/d	28 (14.1%)	15 (17.4%)	12 (15.8%)	
<3 h/d	39 (19.7%)	25 (29.1%)	28 (36.8%) ^c^	
not applicable	131 (66.2%)	46 (53.5%)	36 (47.4%)	
**use of airconditioning**				0.277
>3 h/d	27 (13.6%)	8 (9.3%)	6 (7.9%)	
<3 h/d	47 (23.7%)	16 (18.6%)	13 (17.1%)	
not applicable	124 (62.6%)	62 (72.1%)	57 (75.0%)	
**time of watching TV**				0.313
>3 h/d	97 (49.0%)	45 (52.3%)	30 (39.5%)	
<3 h/d	92 (46.5%)	34 (39.6%)	41 (53.9%)	
not applicable	9 (4.5%)	7 (8.1%)	5 (6.6%)	

*: P<0.05, †: P<0.01.

a: Cch VS DES & non-Cch has statistical significance.

b: DES & non-Cch VS the other two groups has statistical significance.

c: Normal control VS Cch has statistical significance.

The clinical characteristics and ocular surface parameters of each group were highlighted in Table 3. The subjects with Cch had significantly decreased tear film stability, even compared with those with DES. There were no significant differences in the ST values and corneal fluorescence staining scores between participants with Cch and ones with DES.

**Table 3:** The clinical characteristics and ocular surface parameters of enrolled subjects.

	Cch (n = 198)		DES & non-Cch (n = 86)		normal controls (n = 76)		P value
	OD	OS	OD	OS	OD	OS	
**Visual acuity**	0.55±0.21	0.51±0.24	0.58±0.24	0.57±0.23	0.55±0.27	0.54±0.23	0.886
**ST**							<0.001 ‡
<5 mm	79 (39.9%)	80 (40.4%)	28 (32.6%)	30 (34.9%)	0	0	Cch vs DES: 0.051
5–10 mm	63 (31.8%)	59 (29.8%)	21 (24.4%)	17 (19.8%)	25 (32.9%)	23 (30.3%)	Cch vs Con: <0.001 ‡
>10 mm	56 (28.3%)	59 (29.8%)	37 (43.0%)	39 (45.3%)	51 (67.1%)	53 (69.7%)	DES vs Con: <0.001 ‡
**TBUT**							<0.001 ‡
<5 s	95 (48.0%)	92 (46.5%)	28 (32.6%)	28 (32.6%)	0	1 (1.3%)	Cch vs DES: 0.001 †
5–10 s	82 (41.4%)	84 (42.4%)	33 (38.3%)	35 (40.7%)	29 (38.2%)	29 (38.2%)	Cch vs Con: <0.001 ‡
>10 s	21 (10.6%)	22 (11.1%)	25 (29.1%)	23 (26.7%)	47 (61.8%)	46 (60.5%)	DES vs Con: <0.001 ‡
**FS**							0.010 *
binocular positive	3 (1.5%)		1 (1.2%)		0		
monocular positive	3 (1.5%)		4 (4.7%)		0		
medium score	1	1	1	1	0	0	
**IOP (mmHg)**	14.9±2.9	14.6±3.5	16.6±3.2	16.5±2.7	16.2±4.5	14.9±5.7	0.648

Con: normal controls.

*: P<0.05, †: P<0.01, ‡: P<0.001.

The OSDI score, NEI VFQ-25 composite score, and the scores for each VFQ-25 subscale were all presented in Table 4. The OSDI scores of both the Cch and DES groups were significantly higher than those of the normal controls. Meanwhile, the VFQ-25 composite scores of the Cch and DES subjects were significantly lower than those of the normal controls. The similar results were also found concerning the subscale scores for general health and ocular pain. Nevertheless, subscale score for mental health was significantly lower in participants with DES than those with Cch.

**Table 4:** The OSDI score, NEI VFQ-25 composite score, and the scores for each VFQ-25 subscale in enrolled subjects.

	Cch (n = 198)	DES & non-Cch (n = 86)	normal controls (n = 76)	P value	subgroup comparison
OSDI					
average	19.3±22.1	16.8±12.2	3.6±5.2	<0.001^‡^	Cch vs DES: 0.934, Cch vs Con: <0.001, DES vs Con: <0.001
median	12.5	15.9	0		
VFQ-25					
General health	56.3±14.6	56.2±15.2	62.5±15.7	0.012*	Cch vs DES: 0.998, Cch vs Con: 0.013, DES vs Con: 0.004
General Vision	65.9±13.1	68.2±13.7	69.8±12.7	0.096	
Ocular Pain	84.0±19.4	83.6±14.9	93.7±12.8	0.001^†^	Cch vs DES: 0.957, Cch vs Con: 0.001, DES vs Con: <0.001
Near Activities	81.6±17.4	89.1±16.1	87.9±14.1	0.001^†^	Cch vs DES: 0.003, Cch vs Con: 0.027, DES vs Con: 0.951
Distance Activities	91.1±14.6	94.4±12.3	96.3±8.9	0.116	
Social Functioning	93.1±13.5	95.9±11.9	97.7±8.2	0.202	
Mental Health	90.3±15.6	85.2±15.2	92.6±12.2	0.008^†^	Cch vs DES: 0.033, Cch vs Con: 0.878, DES vs Con: 0.011
Role Difficulties	88.2±21.1	91.6±15.5	93.7±16.0	0.096	
Dependency	93.6±15.7	94.4±12.6	96.3±13.9	0.462	
Color Vision	93.7±12.7	95.7±12.4	97.7±8.5	0.054	
Peripheral Vision	92.0±15.8	95.1±12.8	97.3±8.9	0.234	
VFQ-25 composite score	87.4±12.3	87.7±10.0	92.3±8.0	0.007^†^	Cch vs DES: 0.854, Cch vs Con: 0.004, DES vs Con: 0.011

OSDI: Ocular Surface Disease Index, NEI VFQ-25: 25-item National Eye Institute Visual Function Questionnaire, DES: dry eye syndrome, Cch: conjunctivochalasis, Con: normal controls.

*: P<0.05, †: P<0.01, ‡: P<0.001.

Among 198 subjects with Cch, 21.7% were classified as G1, 66.2% as G2, and the rest 12.1% as G3. Further classification based on location, height, gaze-dependent changes, and digital pressure–dependent changes were presented in Table 5. The clinical characteristics and ocular surface parameters of each subgroup were shown in Table S1 in File S1, demonstrating that decreased TBUT was found in participants with higher grade of Cch. Further comparison made within G2 Cch subjects revealed that Cch at middle and nasal sides disturbed the stability of the tear film more so than Cch at the temporal side. Notably, subjects with the following conditions exhibited decreased tear film stability: chalasis folds higher than tear meniscus height, Cch with punctal occlusion, and increased extent of chalasis in downgaze. In addition, the tear production volume was lower in participants either with chalasis folds higher than tear meniscus height or punctal occlusion caused by Cch.

**Table 5:** The classification of participants with Cch.

	1	2	3
Grade (G)	43 (21.7%)	131 (66.2%)	24 (12.1%)
G1-Subgrade	T: 33(76.6%)	M: 5 (11.6%)	N: 5 (11.6%)
G2-Subgrade	T+N: 115 (87.8%) M+N: 16 (12.2%)		
Folds versus Tear Meniscus Height (H)	86 (43.4%)	70 (35.4%)	42 (21.2%)
Punctal Occlusion (O)	172 (86.9%)	26 (13.1%)	
Changes in Down Gaze (DG)	117 (59.1%)	7 (3.5%)	74 (37.4%)
Changes by Digital Pressure (P)	125 (63.1%)	36 (18.2%)	37 (18.7%)

T: Temporal, M:Middle, N: Nasal.

OSDI scores and VFQ-25 subscale scores were further compared among the subgroups of Cch, as shown in Table S2 in 2. The median OSDI scores reported by participants with Cch at the nasal side and those with punctal occlusion were 29.2 and 20 respectively, both of which were significantly higher than those of their counterparts. Meanwhile, significantly lower VFQ-25 composite scores were found in subjects either with chalasis folds higher than tear meniscus height or Cch-induced punctal occlusion, which were 82.1±13.9 and 79.5±19.8 respectively. The VFQ-25 composite score of the subjects with decreased extent of chalasis on digital pressure was 92.0±11.0, which was significantly higher than that of the others. Notably, participants with any one of the following conditions reported a significantly lower subscale score for ocular pain: G3 Cch, chalasis folds higher than tear meniscus height, Cch with punctal occlusion, increased extent of chalasis in downgaze, and increased extent of chalasis on digital pressure. Moreover, the subjects with either with chalasis folds higher than tear meniscus height or punctal occlusion caused by Cch had significantly lower subscale scores for mental health, role difficulties and dependency.

The multiple regression analysis revealed that OSDI score was significantly correlated with the ST value in eyes with a higher grade of Cch (P = 0.004). In addition, age was significantly correlated with the VFQ-25 composite score (P = 0.046).

## Discussion

The present study found that compared with normal controls, those with Cch reported a significantly higher OSDI score and a lower VFQ-25 composite score, denoting an impaired VR-QoL in these subjects. In eyes with Cch, the redundant conjunctiva, which occupies the tear meniscus at the lower lid margin partially or completely, may disrupt the normal function of the tear meniscus such as the reservoir for retaining tears [26], the route for tears along the lid margin [27], and delivery of tears to the ocular surface at the time of blinking. Moreover, redundant conjunctiva may behave like a foreign body, causing irritation to the cornea and/or lid margin. In addition, an ectopic tear meniscus neighboring a redundant conjunctiva produces meniscus-induced tear film thinning [26], [28], leading to precorneal tear film instability. Previous reports confirmed that Cch could lead to decreased tear film stability [11], ocular surface inflammation [29] and increased tear osmolarity [10], causing ocular discomfort symptoms and adverse effects on VR-QoL.

Comparisons between the Cch and DES subjects in the present study showed that both groups had a similar OSDI score and VFQ-25 composite score. Moreover, there were no significant between-group differences in their subscale scores for ocular pain. These findings demonstrate that Cch and DES generally have similar impacts of ocular discomfort on VR-QoL. It is notable that although the subjects with DES are mainly mild to moderate, since the OSDI score is not very high, they reported significantly lower subscale score for mental health than those with Cch or normal controls, demonstrating that DES caused more severe mental impairment than Cch.

The current study found more severe disturbance in the tear film stability of participants with any of the following conditions: a higher grade of Cch, especially Cch at middle and nasal sides, chalasis folds higher than tear meniscus height, Cch with punctal occlusion, and increased extent of chalasis in downgaze. Accordingly, the majority of subjects with these conditions had significantly higher OSDI scores and lower VFQ-25 scores, implying that the degree of impaired VR-QoL has a significant association with the severity of Cch.

The higher grade of Cch means for more areas along the lower lid were involved, causing a subclinical ectropion, as confirmed by FD-OCT [23], and leading to disturbances to tear excretion. Chalasis folds higher than the tear meniscus height suggest that more tear meniscus were occupied by redundant conjunctiva, disrupting the function of normal tear meniscus and interfering tear flow from fornix to tear meniscus [30]. A redundant conjunctiva, especially at the nasal side, can obstruct the inferior puncta and cause punctal obstruction, leading to delayed tear clearance [2], increased tear film and ocular surface inflammation [29], [31], and severe ocular irritation symptoms. Increased extent of chalasis in downgaze or on digital pressure also implies more disruption of the tear meniscus, and may lead to greater tear film abnormalities due to dysfunction of the tear meniscus as mentioned above. All the aforementioned factors disturb the distribution, drainage, and even production of tear, which would be expected to contribute to an adverse VR-QOL.

It should be highlighted that unlike DES, traditional methods to treat tear film abnormalities, such as artificial tears and punctal occlusion, are not effective in Cch [32], and they cannot compensate for the adverse effects of Cch on VR-QoL. Only surgical correction can alleviate symptoms such as tearing and improve signs [11], [32]. However, the invasive surgical manipulations not only exert an economic burden, but also cause further physical injury and a mental burden, adversely affecting the patient’s QoL.

The present study contains two specific limitations. One is that the study mainly focused on a community population rather than a hospital population. It is reasonable to deduce that there will be more symptomatic participants in a hospital population than in a community population, and that they are likely to have more severe signs and report a worse VR-QoL. Potential differences in the VR-QoL of these two populations merit further investigation. The second limitation is that we did not quantitatively evaluate the alterations in tear clearance due to the lack of noninvasive and rapid tests suitable for a large-scale population-based survey. A recent study has reported that anterior segment optical coherence tomography could be applied to evaluate tear clearance [33]. It might be a promising tool to quantitatively measure tear clearance and determine its impact on tear function and VR-QoL.

In summary, Cch had an adverse impact on VR-QoL in a community population, and the impairment in VR-QoL showed a significant correlation with disease severity and tear film abnormalities. With greater life expectancy and ever-increasing demands in elderly people for a good QoL, the popularization of public education on Cch and its impact on daily life cannot be ignored. The findings of the present study add new insights into the consideration of Cch as a public health problem that deserves further study.

## Supporting Information

File S1 **Supporting tables.**
(DOC)
