# Supplementary material for: Impact of Conjunctivochalasis on Visual Quality of Life: A Community Population Survey
Source: PLoS One. 2014 Oct 20;9(10):e110821. doi: 10.1371/journal.pone.0110821 (PMC4203838; doi:10.1371/journal.pone.0110821)
Supplement: File S1 — Supporting tables. (DOC) [file pone.0110821.s001.doc]

Table S1 The clinical characteristics and ocular surface parameters of each subgroup in subjects with Cch

|  | **Visual acuity** | | **IOP (mmHg)** | | **ST** | | | | | | **TBUT** | | | | | | | | **CFS** | | |
| --- | --- | --- | --- | --- | --- | --- | --- | --- | --- | --- | --- | --- | --- | --- | --- | --- | --- | --- | --- | --- | --- |
|  | OD | OS | OD | OS | OD | | | OS | | | OD | | | OS | | | | | binocular positive | monocular positive | |
|  |  |  |  |  | <5mm | 5-10mm | >10mm | <5mm | 5-10mm | >10mm | <5s | 5-10s | >10s | <5s | 5-10s | | >10s | |  |  |  |
| **Grade** |  |  |  |  |  |  |  |  |  |  |  |  |  |  | |  | |  |  |  | |
| G1 (n=43) | 0.51±0.25 | 0.61±0.39 | 16.7±2.9 | 16.8±2.9 | 19(44.2%) | 11(25.6%) | 13(30.2%) | 17(39.6%) | 13(30.2%) | 13(30.2%) | 15(34.9%) | 22(51.2%) | 6(14.9%) | 14(32.6%) | | 23(53.4%) | | 6(14.0%) | 1 | 0 | |
| G2 (n=131) | 0.54±0.22 | 0.55±0.21 | 16.2±3.1 | 16.0±3.1 | 51(38.9%) | 46(35.1%) | 34(26.0%) | 54(41.2%) | 40(30.5%) | 37(28.3%) | 65(49.6%) | 51(38.9%) | 15(11.5%) | 63(48.1%) | | 54(41.2%) | | 14(10.7%) | 1 | 3 | |
| G3 (n=24) | 0.48±0.21 | 0.53±0.26 | 16.2±3.6 | 17.0±3.4 | 9(37.5%) | 6(25.0%) | 9(37.5%) | 9(37.5%) | 6(25.0%) | 9(37.5%) | 15(62.5%) | 9(37.5%) | 0 | 15(62.5%) | | 7(29.2%) | | 2(8.3%) | 1 | 0 | |
| **P value** | **0.064** | | **0.643** | | **0.579** | | | | | | **0.018 *** | | | | | | | | **0.809** | | |
| G1-Subgrade |  |  |  |  |  |  |  |  |  |  |  |  |  |  | |  | |  |  | |  |
| T (n=33) | 0.53±0.26 | 0.63±0.41 | 16.5±2.7 | 16.6±2.8 | 13(39.4%) | 10(30.3%) | 10(30.3%) | 12(36.4%) | 10(30.3%) | 11(33.3%) | 11(33.3%) | 18(55.6%) | 4(12.1%) | 10(30.3%) | | 19(57.6%) | | 4(12.1%) | 1 | | 0 |
| M (n=5) | 0.51±0.21 | 0.63±0.38 | 17.7±2.8 | 16.8±1.5 | 3(60.0%) | 0 | 2(40.0%) | 2(40.0%) | 2(40.0%) | 1(20.0%) | 1(20.0%) | 2(40.0%) | 2(40.0%) | 2(40.0%) | | 2(40.0%) | | 1(20.0%) | 0 | | 0 |
| N (n=5) | 0.27±0.05 | 0.38±0.09 | 18.0±3.8 | 17.2±3.45 | 3(60.0%) | 1(20.0%) | 1(20.0%) | 3(60.0%) | 1(20.0%) | 1(20.0%) | 3(60.0%) | 2(40.0%) | 0 | 2(40.0%) | | 2(40.0%) | | 1(20.0%) | 0 | | 0 |
| **P value** | **0.292** | | **0.920** | | **0.703** | | | | | | **0.438** | | | | | | | | **0.089** | | |
| G2-Subgrade |  |  |  |  |  |  |  |  |  |  |  |  |  |  | |  | |  |  | |  |
| Temporal+Nasal(n=115) | 0.54±0.22 | 0.55±0.21 | 16.2±3.1 | 15.8±3.1 | 43(37.4%) | 40(34.8%) | 32(27.8%) | 46(40.0%) | 33(28.7%) | 36(31.3%) | 54(47.0%) | 49(42.6%) | 12(10.4%) | 53(46.1%) | | 50(43.5%) | | 12(10.4%) | 0 | | 2 |
| Middle+Nasal(n=16) | 0.59±0.25 | 0.57±0.19 | 16.3±2.6 | 17.3±2.6 | 8(50.0%) | 6(37.5%) | 2(12.5%) | 8(50.0%) | 7(43.8%) | 1(6.2%) | 11(68.7%) | 2(12.5%) | 3(18.8%) | 10(62.5%) | | 4(25.0%) | | 2(12.5%) | 1 | | 1 |
| **P value** | **0.809** | | **0.166** | | **0.109** | | | | | | **0.044 *** | | | | | | | | **0.248** | | |
| **Height** |  |  |  |  |  |  |  |  |  |  |  |  |  |  | |  | |  |  | |  |
| H1 (n=86) | 0.59±0.23 | 0.63±0.28 | 16.1±2.9 | 16.0±3.2 | 30(34.9%) | 31(36.0%) | 25(29.1%) | 29(33.7%) | 22(25.6%) | 35(40.7%) | 41(47.7%) | 32(37.2%) | 13(15.1%) | 41(47.7%) | | 33(38.4%) | | 12(13.9%) | 2 | | 1 |
| H2 (n=70) | 0.49±0.22 | 0.53±0.21 | 16.3±3.2 | 16.2±3.1 | 29(41.4%) | 24(34.3%) | 17(24.3%) | 30(42.9%) | 26(37.1%) | 14(20.0%) | 30(42.9%) | 33(47.1%) | 7(10.0%) | 28(40.0%) | | 33(47.1%) | | 9(12.9%) | 1 | | 2 |
| H3 (n=42) | 0.50±0.24 | 0.45±0.20 | 16.8±3.4 | 17.1±2.8 | 20(47.6%) | 8(19.1%) | 14(33.3%) | 21(50.0%) | 11(26.2%) | 10(23.8%) | 24(57.1%) | 17(40.5%) | 1(2.4%) | 23(54.8%) | | 18(42.8%) | | 1(2.4%) | 0 | | 0 |
| **P value** | **0.053** | | **0.541** | | **0.033 *** | | | | | | **0.041 *** | | | | | | | | **0.918** | | |
| **Occlusion** |  |  |  |  |  |  |  |  |  |  |  |  |  |  | |  | |  |  | |  |
| O1 (n=172) | 0.53±0.23 | 0.57±0.27 | 16.3±3.2 | 16.3±3.1 | 64(37.2%) | 56(32.6%) | 52(30.2%) | 64(37.2%) | 52(30.2%) | 56(32.6%) | 76(44.2%) | 75(43.6%) | 21(12.2%) | 73(42.4%) | | 77(44.8%) | | 22(12.8%) | 2 | | 2 |
| O2 (n=26) | 0.49±0.19 | 0.51±0.20 | 16.2±3.0 | 16.3±3.1 | 15(57.7%) | 7(26.9%) | 4(15.4%) | 16(61.5%) | 7(26.9%) | 3(11.6%) | 19(73.1%) | 7(26.9%) | 0 | 19(73.1%) | | 7(26.9%) | | 0 | 1 | | 1 |
| **P value** | **0.176** | | **0.711** | | **0.004 †** | | | | | | **<0.001 ‡** | | | | | | | | **0.995** | | |
| **Down Gaze** |  |  |  |  |  |  |  |  |  |  |  |  |  |  | |  | |  |  | |  |
| DG1 (n=117) | 0.52±0.21 | 0.55±0.22 | 16.3±3.2 | 16.0±3.2 | 44(37.6%) | 43(36.8%) | 30(25.6%) | 45(38.5%) | 38(32.5%) | 34(29.0%) | 65(55.6%) | 42(35.9%) | 10(8.5%) | 60(51.3%) | | 45(38.5%) | | 12(10.2%) | 2 | | 3 |
| DG2 (n=7) | 0.57±0.16 | 0.57±0.19 | 16.0±4.0 | 16.7±3.0 | 4(57.1%) | 1(14.3%) | 2(28.6%) | 4(57.1%) | 1(14.3%) | 2(28.6%) | 3(42.9%) | 4(57.1%) | 0 | 4(57.1%) | | 3(42.9%) | | 0 | 0 | | 0 |
| DG3 (n=74) | 0.55±0.25 | 0.58±0.32 | 16.3±3.0 | 16.7±2.9 | 31(41.9%) | 19(25.7%) | 24(32.4%) | 31(41.9%) | 20(27.0%) | 23(31.1%) | 27(36.5%) | 36(48.6%) | 11(14.9%) | 28(37.9%) | | 36(48.6%) | | 10(13.5%) | 1 | | 0 |
| **P value** | **0.292** | | **0.684** | | **0.259** | | | | | | **0.020 †** | | | | | | | | **0.548** | | |
| **Pressure** |  |  |  |  |  |  |  |  |  |  |  |  |  |  | |  | |  |  | |  |
| P1 (n=125) | 0.53±0.21 | 0.56±0.22 | 16.3±3.2 | 16.0±3.1 | 50(40.0%) | 43(34.4%) | 32(25.6%) | 50(40.0%) | 40(32.0%) | 35(28.0%) | 66(52.8%) | 47(37.6%) | 12(9.6%) | 61(48.8%) | | 52(41.6%) | | 12(9.6%) | 2 | | 3 |
| P2 (n=36) | 0.52±0.22 | 0.55±0.24 | 16.3±3.3 | 17.0±3.0 | 16(44.4%) | 10(27.8%) | 10(27.8%) | 17(47.2%) | 10(27.8%) | 9(25.0%) | 16(44.4%) | 16(44.4%) | 4(11.2%) | 14(38.9%) | | 17(47.2%) | | 5(13.9%) | 0 | | 0 |
| P1 (n=37) | 0.56±0.28 | 0.58±0.38 | 16.1±2.7 | 16.5±3.0 | 13(35.1%) | 10(27.1%) | 14(37.8%) | 13(35.1%) | 9(24.4%) | 15(40.5%) | 13(35.1%) | 19(51.4%) | 5(13.5%) | 17(46.0%) | | 15(40.5%) | | 5(13.5%) | 1 | | 0 |
| **P value** | **0.301** | | **0.362** | | **0.232** | | | | | | **0.442** | | | | | | | | **0.548** | | |

*: P<0.05, †: P<0.01, ‡: P<0.001

T: Temporal, M:Middle, N: Nasal

Table S2 Comparison on OSDI scores, VFQ-25 subscale scores and VFQ-25 composite score among subgroups of Cch

|  |  | OSDI | | VFQ-25 | | | | | | | | | | | VFQ-25 composite score |
| --- | --- | --- | --- | --- | --- | --- | --- | --- | --- | --- | --- | --- | --- | --- | --- |
|  |  | average | median | General health | General Vision | Ocular Pain | Near Activities | Distance Activities | Social Functioning | Mental Health | Role Difficulties | Dependency | Color Vision | Peripheral Vision |  |
| **Grade** | G1 (n=43) | 13.3±11.8 | 10 | 55.4±14.7 | 66.8±13.8 | 83.2±19.7 | 85.9±17.6 | 93.4±11.8 | 94.2±11.3 | 88.9±12.0 | 87.4±19.1 | 94.9±10.9 | 95.5±11.1 | 94.2±12.0 | 88.5±10.5 |
|  | G2 (n=131) | 20.1±23.6 | 13.6 | 56.9±14.6 | 66.3±12.7 | 85.8±18.1 | 80.0±17.7 | 90.5±15.2 | 92.9±14.4 | 90.9±15.8 | 89.0±21.4 | 94.0±16.1 | 93.8±12.8 | 91.4±17.3 | 87.5±12.5 |
|  | G3 (n=24) | 22.7±25.2 | 15 | 54.5±14.1 | 62.1±14.1 | 74.5±22.9 | 84.1±13.2 | 90.8±15.2 | 92.4±11.5 | 89.4±19.2 | 84.8±21.8 | 89.5±19.4 | 90.2±14.3 | 92.4±11.5 | 85.0±13.5 |
| **P value** | | **0.169** | | **0.688** | **0.321** | **0.032 *** | **0.140** | **0.541** | **0.837** | **0.753** | **0.662** | **0.388** | **0.288** | **0.606** | **0.564** |
| G1-Subgrade | T (n=33) | 12.4±10.7 | 10 | 56.8±14.9 | 67.0±13.8 | 83.9±19.4 | 86.9±17.8 | 94.1±11.0 | 94.7±11.1 | 89.6±11.8 | 87.4±20.0 | 95.5±10.7 | 96.2±10.9 | 94.7±11.9 | 89.0±10.0 |
|  | M (n=5) | 7.5±4.7 | 7.5 | 53.8±6.7 | 68.8±15.6 | 78.1±25.6 | 87.5±9.3 | 93.8±10.8 | 93.8±10.8 | 89.1±12.8 | 93.7±10.2 | 93.7±10.3 | 93.8±10.8 | 93.8±10.8 | 88.6±12.3 |
|  | N (n=5) | 26.2±13.1 | 29.2 | 55.5±17.0 | 63.0±9.3 | 65.0±21.5 | 78.3±16.3 | 88.8±15.0 | 95.0±10.0 | 86.3±10.0 | 70.0±24.5 | 90.0±12.2 | 95.0±10.0 | 90.0±12.2 | 82.1±9.8 |
| **P value** | | **0.024 *** | | **0.921** | **0.802** | **0.179** | **0.594** | **0.647** | **0.985** | **0.849** | **0.171** | **0.599** | **0.903** | **0.730** | **0.412** |
| G2-Subgrade | T+N(n=115) | 20.4±25.0 | 12.5 | 56.7±14.4 | 66.2±12.8 | 85.9±18.1 | 77.9±17.8 | 89.3±16.0 | 91.8±15.2 | 90.5±16.8 | 88.2±22.0 | 93.1±17.2 | 92.8±13.5 | 90.4±18.2 | 86.6±13.2 |
|  | M+N(n=16) | 18.0±13.7 | 15 | 57.6±16.4 | 68.1±12.5 | 91.0±10.9 | 93.2±11.5 | 97.2±6.3 | 98.6±5.7 | 93.8±7.2 | 97.9±6.3 | 100±0 | 98.6±5.7 | 97.2±7.9 | 93.6±4.5 |
| **P value** | | **0.687** | | **0.797** | **0.577** | **0.250** | **0.001 †** | **0.042 *** | **0.066** | **0.431** | **0.067** | **0.094** | **0.077** | **0.125** | **0.030 *** |
| **Height** | H1 (n=86) | 18.8±23.6 | 13.1 | 58.3±15.0 | 70.6±10.5 | 91.4±13.2 | 83.8±17.1 | 94.0±11.0 | 95.5±10.4 | 93.6±10.2 | 93.6±16.4 | 97.9±8.6 | 96.0±9.9 | 95.5±11.6 | 91.2±8.0 |
|  | H2 (n=70) | 18.2±22.5 | 11.8 | 54.3±14.2 | 64.0±13.0 | 83.0±17.3 | 79.5±17.6 | 89.9±16.7 | 92.0±16.4 | 89.2±17.0 | 85.5±23.7 | 90.4±18.4 | 92.8±14.1 | 89.3±20.1 | 85.6±14.2 |
|  | H3 (n=42) | 21.1±17.9 | 14.3 | 55.6±13.8 | 59.1±14.6 | 69.4±24.8 | 80.6±17.3 | 86.9±16.2 | 90.0±13.2 | 85.2±20.4 | 80.9±22.4 | 90.0±19.8 | 90.0±14.6 | 89.4±13.6 | 82.1±13.9 |
| **P value** | | **0.804** | | **0.217** | **<0.001 ‡** | **<0.001 ‡** | **0.275** | **0.027 *** | **0.072** | **0.013 *** | **0.003 †** | **0.003 †** | **0.063** | **0.052** | **<0.001 ‡** |
| **Occlusion** | O1 (n=172) | 17.8±21.1 | 12.5 | 57.2±14.5 | 67.0±12.8 | 86.5±17.5 | 82.7±16.6 | 92.2±12.9 | 94.4±11.2 | 91.6±12.8 | 90.3±19.5 | 95.0±13.1 | 94.9±11.1 | 92.4±13.9 | 88.8±10.1 |
|  | O2 (n=26) | 28.8±27.3 | 20 | 49.3±13.7 | 58.0±13.2 | 64.7±22.0 | 73.6±21.1 | 82.6±22.3 | 87.3±22.9 | 80.7±27.1 | 72.3±25.5 | 83.0±26.2 | 88.4±19.1 | 89.5±23.6 | 79.5±19.8 |
| **P value** | | **0.026 *** | | **0.017 *** | **0.006 †** | **<0.001 ‡** | **0.061** | **0.059** | **0.176** | **0.042 *** | **0.004 †** | **0.044 *** | **0.247** | **0.318** | **0.018 *** |
| **Down Gaze** | DG1 (n=117) | 20.3±25.2 | 13.6 | 53.1±14.5 | 65.0±12.0 | 87.1±18.1 | 75.3±16.8 | 89.0±15.6 | 91.5±15.3 | 88.9±16.7 | 87.1±22.2 | 92.4±16.8 | 92.3±13.7 | 90.2±18.2 | 85.9±12.9 |
|  | DG2 (n=7) | 15.1±4.7 | 12.5 | 65.0±12.4 | 62.9±13.1 | 60.7±23.6 | 85.7±9.7 | 95.5±8.7 | 92.9±11.3 | 93.8±11.1 | 82.1±17.5 | 96.4±8.7 | 92.9±11.3 | 92.9±11.3 | 85.6±10.0 |
|  | DG3 (n=74) | 17.5±17.4 | 12.5 | 60.6±13.4 | 67.7±14.6 | 81.4±19.0 | 91.3±14.1 | 94.0±12.8 | 95.8±9.7 | 92.3±13.7 | 90.5±19.3 | 95.4±14.1 | 96.0±10.9 | 94.9±10.8 | 89.9±11.1 |
| **P value** | | **0.628** | | **0.001 †** | **0.305** | **<0.001 ‡** | **<0.001 ‡** | **0.052** | **0.098** | **0.284** | **0.411** | **0.394** | **0.156** | **0.127** | **0.080** |
| **Pressure** | P1 (n=125) | 19.2±24.3 | 12.5 | 56.3±14.3 | 65.7±12.1 | 86.9±17.3 | 76.8±17.3 | 89.7±15.3 | 92.2±14.9 | 89.5±16.3 | 86.7±23.0 | 92.9±16.2 | 93.0±13.3 | 90.6±17.8 | 86.4±12.6 |
|  | P2 (n=36) | 20.7±19.9 | 13.1 | 59.5±15.0 | 63.1±13.4 | 71.9±22.9 | 85.6±16.0 | 90.5±13.6 | 92.0±12.6 | 91.7±13.2 | 87.2±18.5 | 93.8±14.6 | 92.4±12.9 | 91.7±13.2 | 86.0±11.6 |
|  | P3 (n=37) | 16.8±15.9 | 11.1 | 60.2±13.7 | 69.5±15.4 | 86.0±17.7 | 93.7±11.0 | 94.6±11.5 | 97.3±7.2 | 91.9±15.2 | 94.2±14.9 | 95.9±14.8 | 97.3±9.7 | 97.3±7.8 | 92.0±11.0 |
| **P value** | | **0.747** | | **0.303** | **0.115** | **<0.001 ‡** | **<0.001 ‡** | **0.054** | **0.114** | **0.613** | **0.158** | **0.595** | **0.157** | **0.076** | **0.041 *** |

*: P<0.05, †: P<0.01, ‡: P<0.001

OSDI: Ocular Surface Disease Index, NEI VFQ-25: 25-item National Eye Institute Visual Function Questionnaire, Cch: conjunctivochalasis, T: Temporal, M:Middle, N: Nasal
